# Association of Single Nucleotide Polymorphisms in the Lens Epithelium-Derived Growth Factor (LEDGF/p75) with HIV-1 Infection Outcomes in Brazilian HIV-1+ Individuals

**DOI:** 10.1371/journal.pone.0101780

**Published:** 2014-07-21

**Authors:** Caroline Pereira Bittencourt Passaes, Cynthia Chester Cardoso, Diogo Gama Caetano, Sylvia Lopes Maia Teixeira, Monick Lindenmeyer Guimarães, Dayse Pereira Campos, Valdilea Gonçalves Veloso, Dunja Z. Babic, Mario Stevenson, Milton Ozório Moraes, Mariza Gonçalves Morgado

**Affiliations:** 1 Laboratório de AIDS e Imunologia Molecular, IOC/FIOCRUZ, Rio de Janeiro, Brasil; 2 Laboratório de Virologia Molecular Animal, Departamento de Genética, Universidade Federal do Rio de Janeiro, Rio de Janeiro, Brasil; 3 Instituto de Pesquisa Clínica Evandro Chagas, FIOCRUZ, Rio de Janeiro, Brasil; 4 Division of Infectious Diseases, Department of Medicine, Miller School of Medicine, University of Miami, Miami, United States of America; 5 Laboratório de Hanseníase, IOC/FIOCRUZ, Rio de Janeiro, Brasil; Institute of Infection and Global Health, United Kingdom

## Abstract

The lens epithelium-derived growth factor p75 (LEDGF/p75), coded by the *PSIP1* gene, is an important host co-factor that interacts with HIV-1 integrase to target integration of viral cDNA into active genes. The aim of this study was to investigate the association of SNPs in the *PSIP1* gene with disease outcome in HIV-1 infected patients. We performed a genetic association study in a cohort of 171 HIV-1 seropositive Brazilian individuals classified as rapid progressors (RP, n = 69), typical progressors (TP, n = 79) and long-term nonprogressors (LTNP, n = 23). The exonic SNP rs61744944 and 9 *tag* SNPs were genotyped. A group of 192 healthy subjects was analyzed to determine the frequency of SNPs and haplotypes in the general population. Linkage disequilibrium (LD) analyses indicated that the SNPs analyzed were not in high LD (r^2^<0.8). Logistic regression models suggested that patients carrying the T allele rs61744944 (472L) were more likely to develop a LTNP phenotype (OR = 4.98; p = 0.05) as compared to TP group. The same trend was observed when LTNPs were compared to the RP group (OR = 3.26). Results of haplotype analyses reinforced this association, since the OR values obtained for the haplotype carrying allele T at rs61744944 also reflected an association with LTNP status (OR = 6.05; p = 0.08 and OR = 3.44; p = 0.12 for comparisons to TP and RP, respectively). The rare missense variations Ile436Ser and Thr473Ile were not identified in the patients enrolled in this study. Gene expression analyses showed lower LEDGF/p75 mRNA levels in peripheral blood mononuclear cells obtained from HIV-1 infected individuals. However, these levels were not influenced by any of the SNPs investigated. In spite of the limited number of LTNPs, these data suggest that the *PSIP1* gene could be associated with the outcome of HIV-1 infection. Further analyses of this gene may guide the identification of causative variants to help predict disease course.

## Introduction

The clinical course of HIV-1 infection is characterized by a symptomatic acute phase, followed by an asymptomatic period with ongoing viral replication and gradual loss of CD4 T cells [1]. In the absence of antiretroviral therapy, HIV-1 infected patients develop immunodeficiency and succumb to AIDS-related diseases. The duration of the asymptomatic period is variable. Most HIV-1 infected individuals (70–80%) progress to AIDS within 5 to 10 years after infection and are classified as typical progressors. Some HIV-1 infected individuals (10–15%) develop AIDS in 2 to 3 years and are classified as rapid progressors. Durable control of viral replication and preservation of CD4^+^ T cells for more than 10 years after infection is observed in a subset of HIV-1 infected individuals (5–15%) commonly referred to as long-term nonprogressors [2], [3]. These differences in the clinical outcomes of HIV-1 infection have been associated with viral factors [4]–[8], innate and adaptive immune responses [9]–[21], and host genetics [22]–[28].

HIV-1 requires cellular co-factors at various stages in its replication cycle. The identification of host proteins interacting with HIV-1 may guide not only the development of novel antiretroviral drugs, but also the identification of new candidate genes associated with different disease outcomes. The lens epithelium-derived growth factor p75 (LEDGF/p75) is a cellular co-factor that interacts with HIV-1 integrase (IN) [29]. LEDGF/p75 is encoded by the *PSIP1* gene, which also encodes the splicing variant p52 [30]. Both variants contains an N-terminal region of 325 residues that harbors PWWP (1–93) and AT hook (178–197) domains, as well as a nuclear localization signal (NLS, 148–156) [31]. The larger variant (p75) interacts with HIV-1 integrase through a region at the C-terminus called integrase binding domain (IBD, 347–429) [31]. A small number of residues are involved in the interaction between LEDGF/p75 IBD (K364, I365, D366, F406, V408) and HIV-1 IN (E69, A129, W132, V165, R166, Q168) [31], [32]. During HIV-1 replication, LEDGF/p75 plays an important role in mediating tethering of the viral genome to host chromatin, facilitating the integration into transcription units, being important for the HIV integration site distribution [33]–[38].

As integration of the viral genome is a prerequisite for HIV-1 replication, genetic variations in LEDGF/p75 could lead to different disease outcomes. Some studies have suggested that genetic variation in *PSIP1* may influence the susceptibility to HIV-1 infection and disease progression. However, these results need to be validated in different populations [39]–[41]. In the current study, we performed a comprehensive analysis of the genetic variations in *PSIP1* in a cohort of Brazilian HIV-1 infected individuals with different disease outcomes.

## Materials and Methods

### Ethics Statement

The present study was approved by the IPEC/FIOCRUZ Institutional Review Board (IRB), as well as by the Brazilian National Commission of Ethics in Research (151/01, 81/2008 and CONEP 14430). The LTNPs patients have signed the written informed consent. For the others patients, the use of stored biological samples was approved by the IPEC IRB for an anonymous unlinked study. In all cases, personal identifications were excluded to ensure patient's anonymity. The present study has not been submitted or accepted for publication elsewhere.

### Subjects and study design

The present study was conducted to characterize the frequencies of *PSIP1* SNPs as well as to establish possible associations between these SNPs and AIDS progression profiles in a cohort of 171 HIV-1 infected Brazilian subjects. Patients were classified as rapid progressors (RP, n = 69), typical progressors (TP, n = 79) and long-term nonprogressors (LTNP, n = 23) according to clinical and laboratory information obtained from the database provided by the Statistics and Document Service of Instituto de Pesquisa Clínica Evandro Chagas (IPEC, Rio de Janeiro). The time of progression to AIDS was based on the time between HIV-1 infection and the occurrence of the first AIDS-defining event (CD4+ T cell counts <350 cells/mm3, occurrence of AIDS-defining disease, use of antiretroviral therapy or death related to AIDS). The criteria to classify these patients were as follows: a) RP are those patients who progressed to AIDS up to 3 years after HIV-1 infection (the date of HIV-1 infection was estimated as the mean between the last negative and the first positive HIV-1 serology, with a maximum interval of 1.5 year between the two tests); b) TP are those patients who progressed to AIDS within 4 to 10 years after HIV-1 infection (the date of HIV-1 infection was estimated as the mean between the last negative and the first positive HIV-1 serology; in the absence of a negative serology the date of infection was inferred to be 6 months before the first positive HIV-1 serology); and c) LTNP are those patients who maintained CD4+ T cell counts >500 cells/mm^3^ without the use of antiretroviral therapy and AIDS-defining events for more than 10 years after HIV-1 infection. In addition, a group of 192 HIV-1 negative individuals from the same geographic region was analyzed. This group was used as population control to characterize the frequency of *PSIP1* SNPs and haplotypes. Therefore, these samples were not included in the association analysis.

In order to assess LEDGF/p75 mRNA expression in LTNPs, a group of 15 HIV-1+ untreated patients with viral loads higher than 10,000 copies/ml and a group of 20 uninfected healthy donors were included as controls. The level of LEDGF/p75 mRNA was quantified for 16 out of 23 LTNPs.

### DNA extraction and SNP genotyping

**Figure 1:**
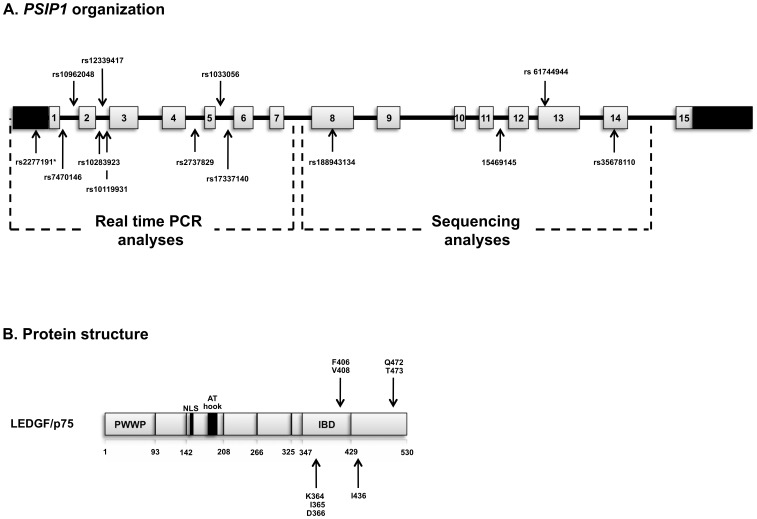
LEDGF/p75 genomic organization and structure. A) *PSIP1* gene organization: Exons are represented as grey blocks and numbered according to their position in the chromossome. 5′ UTR and 3′UTR are indicated by black blocks. Introns are represented by black lines. The SNPs analyzed in the present study are indicated by arrows according to their genomic position. *rs2277191 is located in a non coding region between the two *PSIP1* 5′UTR regions. B) Protein structure: LEDGF/p75 protein domains are represented: PWWP (proline-tryptophan-tryptophan-proline domain); NLS (nuclear localization signal); AT hook (adenine-thymine rich DNA binding region) and IBD (integrase binding domain). The positions associated to the interaction with HIV-1 integrase (K364, I365, D366, F406, V408) and the positions I436 and I473 previously described to present rare missense mutations in LTNPs (Ballana et al., 2012) were investigated in this study.

DNA samples were extracted from 200 µl of whole blood or from isolated PBMCs using QIAamp DNA kit (Qiagen Inc., CA, USA), according to the manufacturer's protocol. *PSIP1* exons 8–14 were amplified and sequenced in order to identify the occurrence of missense mutations and other genetic variations within and outside the exons coding for the IBD. The positions K364, I365, D366, F406 and V408, associated with the interaction of HIV-1 IN and I436 and T473, identified in LTNPs, were investigated. The SNPs rs61744944 (Q472L), rs35678110 and one SNP located in intron 11 identified by Ballana and colleagues [39] (chromosome position 15469145 according to contig NT_008413.18) were also analyzed. A representation of *PSIP1* genomic organization and LEDGF protein structure, as well as all the genetic markers evaluated in the present study, is depicted in Figure 1. PCR reactions were carried out with 100 ng of genomic DNA, 0.25 µM of each specific primer (designed using Primer3 tool; Table S1), 1.25 U of Taq DNA polymerase (Promega, WI, USA), 0.3 mM of each dNTP (Invitrogen, CA, USA) and 1 mM of MgCl_2_ in a final reaction volume of 50 µL. Cycling conditions were as follows: 95°C for 2 minutes followed by 30 cycles of 95°C (30 seconds), 58–60°C (30 seconds) and 72°C (1∶30 minute) and 10 minutes at 72°C for final extension. Samples were purified and sequenced using BigDye Terminator Cycle Sequencing Reaction Kit (Life Technologies, CA, USA). Exons 8, 9, 10 and 14 were characterized for all LTNPs and 30 out of 69 RP, while exons 11–13 were sequenced for all HIV-1+ patients and healthy subjects.

In order to increase gene coverage, a total of 8 *tag* SNPs (rs7470146, rs10962048, rs10283923, rs12339417, rs10119931, rs2737829, rs1033056, rs17337140) was also selected from HapMap data bank using data from CEU population (minor allele frequency of at least 0.05 and r^2^≥0.8 as linkage disequilibrium cutoff). The SNP rs2277191 [40] was also included in this analysis, as depicted in Figure 1. Genotyping of tag SNPs was performed using Real time SNP Genotyping Assays (Table S2), according to the manufacturer's instructions (Life Technologies, CA, USA). Real-time PCR reactions were performed using StepOne and ABI Prism 7500 (Life Technologies, CA, USA) thermal cyclers. PCR reactions were repeated at least once for all the samples with inconclusive results.

### LEDGF/p75 mRNA expression

RNA was isolated from 10 million frozen PBMCs using Trizol reagent, according to the manufacturer's protocol (Life Technologies, CA, USA). All RNA samples were assessed for quality and quantity by Synergy 2 multi-mode plate reader (BioTek Instruments, VT, USA), and RNA concentrations were further used to set up reactions. RNA (1 µg) was treated with DNase I (Life Technologies, CA, USA) in a 10 µl volume and then reverse transcribed using 20 µl reactions of Superscript II (Life Technologies, CA, USA) and Oligo(dT) (0.5 µg/µl). LEDGF/p75 mRNA levels were quantified using a Real Time Gene Expression Assay (Hs00253515_m1), according to manufacturer's protocol. *YWHAZ* (Hs03044281_g1) [42] and *PGK1* (Hs00943178_g1) [43] were used as reference genes for these analyses. Reactions were carried out in an ABI Prism 7500 (Life Technologies, CA, USA) thermal cycler. After PCR, the variation of threshold cycles (ΔCt) between LEDGF/p75 and the mean between the two reference genes (Ct _reference_  =  (Ct *YWHAZ* + Ct PGK1)/2) was calculated for all samples (Ct _LEDGF/p75_ – Ct _reference_). Relative LEDGF/p75 levels are shown as 2^−ΔCt^ values, which represent the fold increase in LEDGF/p75 mRNA expression.

### Statistical analyses

#### SNP genotyping data

All statistical analyses were carried out as previously described [44]. Frequencies of each genotype, allele and minor allele carriers were determined by direct counting. Deviations from Hardy–Weinberg equilibrium (HWE) were accessed by Fisher exact test and pairwise linkage disequilibrium (LD) patterns were determined using the r^2^ statistics. Both analyses were performed using data from healthy subjects. Frequencies of each SNP were compared between patients and healthy subjects using chi-squared tests to assess whether there was any differential profile among HIV+ subjects. Comparisons of patients with different AIDS progression phenotypes were performed by unconditional logistic regression models using frequencies of genotypes, alleles and minor allele carriers. Odds ratios (ORs) were used as effect estimates. Haplotype frequencies were estimated by maximum likelihood and compared between the different patients groups as well. No adjustments for multiple comparisons were applied. All analyses were performed using R for windows version 2.12.1 (R Development Core Team 2010), and the packages “genetics” and “haplo.stats”.

#### Gene expression data

Comparisons of LEDGF/p75 mRNA mean levels in HIV negative individuals, non-treated HIV-1+ and LTNP groups were performed by using Kruskal-Wallis followed by Dunn's post hoc test. Mann-Whitney tests were applied to compare the means of LEDGF/p75 production between minor allele carriers and non-carriers for each SNP. All analyses were performed using the software GraphPad Prism for Windows (version 6.2).

## Results

### Frequencies of *PSIP1* SNPs in healthy subjects and HIV-1+ patients

The frequencies of *PSIP1* SNPs were first described on healthy subjects and then compared to HIV-1+ patients, independent of progression profile, in order to identify possible associations with HIV-1 infection (Table S3). The general characteristics such as age, gender and ethnicity of HIV-1 positive patients and healthy subjects are summarized in Table 1. The mean age of the HIV-1 positive and negative individuals included in the study was approximately 33–34 years old. The distribution of both genders as well as the three classes of self-reported ethnicity (white, mestizoes and blacks) was also similar in both groups.

**Table 1:** General characteristics of the subjects according to the study group and clinical classification.

		Study group		Clinical classification		
Variables		HIV− (N = 192)	HIV+ (N = 171)	LTNP (N = 23)	TP (N = 79)	RP (N = 69)
**Age (mean ± standard deviation)** *		34±9	33±9	33±12	33±9	34±9
**Gender** **	**Female**	54 (0.28)	60 (0.35)	14 (0.61)	19 (0.28)	27 (0.34)
	**Male**	138 (0.72)	111 (0.65)	9 (0.39)	50 (0.72)	52 (0.66)
**Ethnicity** **	**White**	109 (0.57)	100 (0.58)	13 (0.56)	40 (0.51)	47 (0.68)
	**Mestizoes**	53 (0.28)	42 (0.25)	5 (0.22)	26 (0.33)	11 (0.16)
	**Blacks**	28 (0.15)	29 (0.17)	5 (0.22)	13 (0.16)	11 (0.16)

*Age at sample collection for population controls (HIV−) and at first positive test for HIV+ patients.

**Results are shown as N (frequency).

***p<0.05 for ethnicity comparisons according to χ^2^ test. LTNP  =  long-term nonprogressors, TP  =  typical progressors and RP  =  rapid progressors.

We first investigated the occurrence of rare and new mutations in the *PSIP1* exons 8–14 and boundaries (chromosome positions 15474384 to 15466523), as well as substitutions in functional residues in healthy subjects and HIV-1 infected individuals. After direct sequencing, no novel mutations were detected and no differences were observed in exons 8–10 between healthy and HIV-1+ subjects. The rare missense variations Ile436Ser and Thr473Ile were not identified, and the residues K364, I365, D366 and F406 were not mutated in the individuals enrolled in this study. The V408I mutation was identified in one HIV-1+ subject(classified as LTNP). Similarly, the SNP rs35678110 was identified in only one HIV-1+ patient (classified as rapid progressor). Among the 37 exon variations documented in dbSNP in the region spanning exons 8–14, the SNP rs61744944 was the only major variation observed in our samples. The new intron SNP (chromosome position 15469145, according to contig NT_008413.18) was also absent among our cases and healthy controls.

After genotyping all tags (rs7470146, rs10962048, rs10283923, rs12339417, rs10119931, rs2737829, rs1033056, rs17337140) and candidate (rs2277191, rs61744944) SNPs, data from the healthy controls were analyzed separately for quality control and also to determine the distribution of the 10 *PSIP1* polymorphisms in the general population. All the genetic variations investigated in this study were present in healthy Brazilian subjects, with minor allele frequencies ranging from 0.01 (rs61744944) to 0.34 (rs7470146). The rs2277191 variant was also underrepresented (0.02). The frequencies of each genotype, allele and minor allele carriers are shown in Table S3. Two SNPs with previously reported association with AIDS phenotypes (rs12339417 and rs1033056) were excluded from further analysis due to deviations from HWE. Results of pairwise LD analyses showed no definite association between SNPs using a r^2^>0.8 cutoff. Therefore, the remaining 8 SNPs were genotyped in the patients group and used for haplotype analyses (Table S4).

A total of 24 *PSIP1* haplotypes were found in our samples, including both HIV-1 negative and positive subjects (Table S4). From the 15 haplotype combinations detected among HIV-1 positive individuals, 7 were absent in the healthy subjects group. Comparisons including the four haplotypes with minor frequencies of at least 0.03 did not show differences between HIV+ and HIV− groups (Table S4).

### Association between *PSIP1* SNPs and AIDS progression phenotypes

The HIV-1+ individuals were further analyzed according to their progressor status (RP, TP or LTNP), as outlined in Material and Methods. The frequencies of *PSIP1* SNPs and haplotypes were compared among the groups to determine a possible association of *PSIP1* gene variations with AIDS progression profiles. Results of univariate logistic regression models showed an association between allele T at rs61744944 (Q472L) and an LTNP phenotype, with an OR value of 4.98 and a *borderline* p-value of 0.05 when compared to TP under a codominant model (Table 2). The same trend was observed when LTNP patients were compared to RP (OR = 3.26). Analyses of the other 7 SNPs did not show any clear pattern of association with AIDS progression phenotypes (Table 2). Despite the lack of statistical significance, results of haplotype analyses reinforce the association between SNP rs61744944 and LTNP phenotype, since the single haplotype carrying the allele T showed OR values of 6.05 (p = 0.08) and 3.44 (p = 0.12) in comparison to TP and RP phenotypes, respectively (Table 3).

**Table 2:** Association between *PSIP1* SNPs and AIDS progression.

SNP	Genotype	LTNP *vs* TP	RP *vs* TP	LTNP *vs* RP
**rs61744944**	**AA**	reference	reference	reference
	**AT+TT**	4.98 (1.03–24.19; p = 0.05)	1.53 (0.33–7.09; p = 0.59)	3.26 (0.74–14.31; p = 0.12)
**rs17337140**	**GG**	reference	reference	reference
	**GA+AA**	0.22 (0.03–1.82; p = 0.16)	0.97 (0.39–2.37; p = 0.94)	0.23 (0.03–1.90; p = 0.17)
**rs2737829**	**CC**	reference	reference	reference
	**CG+GG**	0.56 (0.06–5.09; p = 0.61)	1.03 (0.28–3.77; p = 0.96)	0.54 (0.06–4.92; p = 0.59)
**rs10119931**	**AA**	reference	reference	reference
	**AC+CC**	1.34 (0.42–4.26; p = 0.61)	1.27 (0.56–2.86; p = 0.56)	1.06 (0.33–3.34; p = 0.92)
**rs10283923**	**CC**	reference	reference	reference
	**CG+GG**	2.01 (0.79–5.15; p = 0.14)	0.83 (0.42–1.61; p = 0.58)	2.44 (0.93–6.38; p = 0.07)
**rs10962048**	**GG**	reference	reference	reference
	**GA+AA**	0.4 (0.08–1.90; p = 0.25)	0.4 (0.15–1.10; p = 0.07)	1 (0.19–5.34; p = 1)
**rs7470146**	**GG**	reference	reference	reference
	**GC+CC**	0.99 (0.39–2.51; p = 0.98)	1.49 (0.78–2.85; p = 0.23)	0.66 (0.26–1.71; p = 0.40)
**rs2277191**	**GG**	reference	reference	reference
	**GA+AA**	n.d.	0.56 (0.10–3.15; p = 0.51)	n.d.

Results are shown as OR (95% confidence interval; p-value) estimated under a codominant model. LTNP  =  long-term nonprogressors, TP  =  typical progressors and RP  =  rapid progressors. n.d  =  not determined.

**Table 3:** Association between *PSIP1* haplotypes and AIDS progression.

rs61744944/rs17337140/rs2737829/rs10119931/rs10283923/rs10962048/rs7470146/rs2277191	LTNP *vs* TP	RP *vs* TP	LTNP *vs* RP
**A/G/C/A/C/G/G/G**	reference	reference	reference
**A/G/C/A/C/G/C/G**	0.52 (0.20–1.40; p = 0.20)	0.98 (0.51–1.86; p = 0.95)	0.79 (0.30–2.05; p = 0.63)
**A/A/C/A/C/G/G/G**	0.13 (0.01–1.33; p = 0.09)	1.00 (0.35–2.86; p = 0.99)	0.24 (0.03–2.21; p = 0.21)
**A/G/C/A/G/A/G/G**	0.10 (0.01–1.07; p = 0.06)	0.30 (0.07–1.30; p = 0.11)	1.56 (0.13–18.5; p = 0.72)
**A/G/C/A/G/G/G/G**	2.61 (0.50–13.6; p = 0.26)	0.58 (0.12–2.72; p = 0.49)	4.53 (0.67–30.5; p = 0.12)
**A/G/C/C/G/G/G/G**	0.27 (0.04–1.85; p = 0.19)	1.07 (0.37–3.11; p = 0.90)	0.46 (0.08–2.56; p = 0.38)
**T/G/C/C/G/G/G/G**	6.05 (0.83–43.9; p = 0.08)	n.d.	3.44 (0.74–15.9; p = 0.12)

Results are shown as OR (95% confidence interval; p-value). LTNP  =  long-term nonprogressors, TP  =  typical progressors and RP  =  rapid progressors. n.d.  =  not done.

### LEDGF/p75 mRNA expression analyses

**Figure 2:**
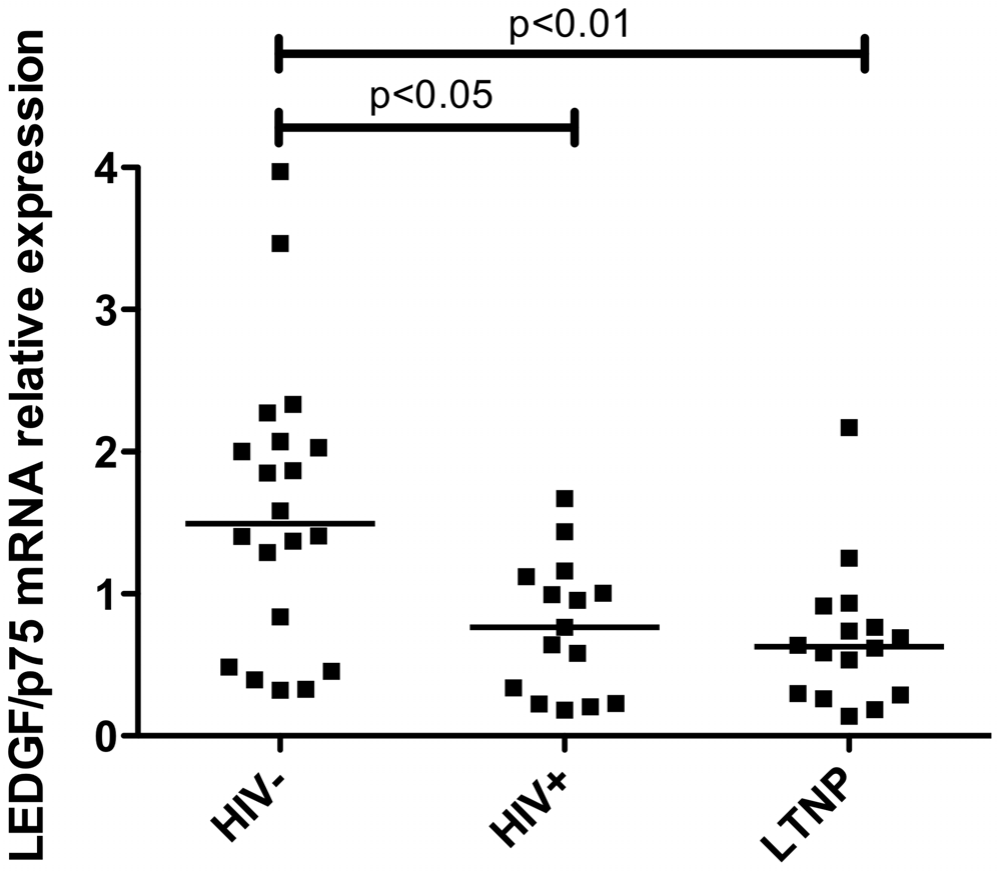
LEDGF/p75 mRNA expression. Expression of LEDGF/p75 in PBMCs from HIV- healthy donors, HIV+ untreated patients with viral load higher than 10,000 copies/ml and LTNPs. Comparisons between groups were performed by Kruskal-Wallis followed by Dunn's post test.

LEDGF/p75 expression was significantly reduced in both untreated HIV-1+ (0.77±0.47; p<0.05) and LTNP (0.69±0.50; p<0.01) groups relative to uninfected individuals (1.59±1), as depicted in Figure 2. No differences were observed between the untreated HIV-1 infected individuals and the LTNPs groups. In order to investigate a putative impact of the SNPs in the modulation of LEDGF/p75 gene expression in LTNPs, we further compared the relative expression between the minor allele carriers and non-carriers of each SNP. No differences in LEDGF/p75 expression levels were observed in LTNPs with distinct genetic background for all SNPs analyzed (data not shown).

## Discussion

The present study aimed to characterize the genetic diversity of *PSIP1* among Brazilian HIV-1 infected individuals and to investigate the association between these markers with AIDS progression. The exonic variations may have an important impact on protein function. The missense variation Q472L (rs61744944; A>T) is located in *PSIP1* exon 13, in a region adjacent to the IBD. Our data show a trend towards an association between the T allele (472L) and LTNP status, suggesting that this variat might confer a protective effect. This SNP has not been reported to significantly affect LEDGF/p75 structure [41], IN binding affinity or HIV replication levels [40], [45]. However, data reported by Madlala and colleagues [40] suggested an association between rs61744944 T and lower viral loads in acute phase of HIV-1 infection. This, together with the results described in the present study, suggest that this variation may have a subtle effect and, as such, would require a larger sample size to reach statistical significance. Alternatively, this SNP might be tagging the effect of a rare variant located in a functional region. Sequencing analysis did not show any other variation in the IBD or its boundaries. However, we cannot rule out the possibility that the SNP rs61744944 might be linked to a rare mutation in a regulatory region.

Resistance to HIV-1 infection and AIDS progression is a complex phenotype in which host genetics plays an important role. The first protective variation to be reported was the Δ32 deletion in *CCR5* gene coding region, followed by several candidate genes studies which described associations with other restriction factors such as CCR5 ligands and HLA alleles, especially class I [46]. The first genome-wide association study (GWAS) was developed to characterize factors associated with control of HIV-1 [23]. Despite the potential of GWAS, new candidates have not been identified. Aside from HLA class I genes HLA-B and HLA-C that were associated with AIDS related outcomes such as control of viremia and LTNP phenotype [22], [47], genes encoding known HIV-1 restriction factors were not associated with disease progression in those studies. Limitations such as the low power of GWAS to detect subtle effects may explain these results. Moreover, rare variations that might play a key role in outcomes such as the LTNP phenotype are also excluded from this approach. Therefore, the complete characterization of the genetic influence on complex phenotypes such as AIDS progression requires the concomitant development of genome-wide as well as candidate genes/regions designs as described in the present study.

The interaction between LEDGF/p75 and HIV-1 IN has emerged as a promising new therapeutic target. Inhibitors of LEDGF/p75-IN interaction (LEDGINs) might be considered for patients failing the currently available drug regimens [48]. In spite of the apparent stability of cellular targets, host genetic variations may impact not only antiviral therapy, but also may be involved in the disease pathogenesis and outcome.

To date, a total of 3 studies have focused on the identification of *PSIP1* polymorphisms in HIV-1 infected individuals [39]–[41]. Two studies were conducted with LTNPs to identify rare mutations associated with this phenotype [39], [41]. According to the data obtained from the control population, all the genetic variations investigated in the present study were present in Brazilian subjects, with allele frequencies similar to those observed in Caucasians (CEU) and Africans (AFR) according to HapMap and 1000 Genomes Projects [49], [50]. Notably, the frequencies of SNPs rs10119931, rs2277191 and rs7470146 were close to the mean between those reported for CEU and AFR populations, reflecting the miscegenation in the Brazilian population [51].

SNP rs2277191 has been previously associated with HIV-1 acquisition and disease progression. Lower CD4^+^ T cell counts and a rapid decline of these cells were observed during the early phase of HIV-1 infection in South African women carrying this particular SNP [40]. Here, we observed similar frequencies of this SNP for both HIV-1 positive and negative individuals. However, we were unable to test the association between this SNP and disease progression due to the small number of subjects carrying allele A.

We have also investigated whether the positions K364, I365, D366, F406, V408, associated with the interaction of HIV-1 IN, were mutated in our cohort. Mutations I365, D366, or F406 disrupt LEDGF/p75-IN interaction and K364 or V408 result in an intermediate phenotype [31], [32]. The V408 was the only position mutated in one LTNP patient. This change affects, but not abolish the interaction with IN [52]. In this particular case, the substitution was V408I, thus the hydrophobic side chain characteristic of this region of the IBD was maintained. A possible impact of this mutation and an association with the nonprogressor status should be further investigated in other LTNP and elite controlers cohorts. The two rare mutations I436S and T473I identified by Ballana and colleagues [39] were not identified in the patients enrolled in this study.

The literature data regarding *PSIP1* variations and gene expression are still controversial. Madlala and colleagues [40] identified an association of the SNP rs12339417 with reduced levels of LEDGF/p75, while Messiaen [41] did not find any differences between mRNA LEDGF/p75 levels and disease progression or CD4 decline and viral load. According to our data, the levels of LEDGF/p75 mRNA produced by the LTNPs of our cohort were not significantly influenced by any of the SNPs investigated. However, we did observe a reduction in the LEDGF/p75 expression in both untreated HIV-1+ and LTNP groups relative to HIV- individuals. These results are in agreement with previous reports [40], [53]. As demonstrated by Mous and colleagues [53], untreated HIV-1 infected individuals have significantly lower levels of LEDGF/p75 and higher levels of APOBEC3G, TRIM5α and tetherin than healthy controls, which might suggest a balance of host innate mechanisms to limit HIV replication by increasing the expression of antiviral restriction factors and decreasing the expression of factors that favor viral replication. In our study, no differences were observed between untreated HIV+ individuals and LTNPs suggesting that the LTNP status is not associated with a lower level of LEDGF/p75. Mous and colleagues [53] showed that while mRNA LEDGF/p75 expression was reduced in PBMCs, elevated protein levels were detected in monocytes relative to uninfected individuals. The mechanisms associated with the regulation of LEDGF/p75 expression in different cell types need to be investigated in HIV-1 infected individuals with different disease outcomes in order to elucidate the association of LEDGF expression with control of HIV-1 infection.

In conclusion, results of the present work reinforce the association of *PSIP1* gene and rs61744944 SNP with LTNP status. Since this variation did not alter the protein function [40], [45], [54], the mechanisms involved in this protection need to be determined. Further studies with larger cohorts of patients with distinct disease outcomes are needed to confirm these trends and to clarify the role of these genetic variations in the function of LEDGF/p75, a pontential target for anti-HIV therapy.

## Supporting Information

Table S1 **Primers to amplify and sequence** ***PSIP1*** **exons 8–14.** The table describes the sequence along with annealing temperatures of each primer and fragment sizes.
(DOCX)

Table S2 **Primers and probes used for** ***PSIP1*** **candidate SNPs genotyping.**
(DOCX)

Table S3 **Frequencies of each genotype, allele and minor allele carriers of** ***PSIP1*** **SNPs and haplotypes in in population controls and HIV^+^ patients.**
(DOCX)

Table S4 **Frequency of** ***PSIP1*** **haplotypes in HIV+ patients and population controls.**
(DOCX)
