# Supplementary material for: Association of Single Nucleotide Polymorphisms in the Lens Epithelium-Derived Growth Factor (LEDGF/p75) with HIV-1 Infection Outcomes in Brazilian HIV-1+ Individuals
Source: PLoS One. 2014 Jul 21;9(7):e101780. doi: 10.1371/journal.pone.0101780 (PMC4105638; doi:10.1371/journal.pone.0101780)
Supplement: Table S1 — Primers to amplify and sequence PSIP1 exons 8–14. The table describes the sequence along with annealing temperatures of each primer and fragment sizes. (DOCX) [file pone.0101780.s001.docx]

**Table S1:** Primers to amplify and sequence *PSIP1* exons 8-14.

| **Exon** | **Intended use** | **Primer sequences (5’ > 3’)** | **Annealing temperature** | **Fragment size (bp)** |
| --- | --- | --- | --- | --- |
| EXON 8 | PCR and sequencing | Foward: tgacagaattccttttatgattgg  Reverse: cacagcgagactccatctca | 59ºC | 550 |
| EXON 9 | PCR and sequencing | Foward: aacaagatgattatttcacggtca  Reverse: ctgcctcagtcaatttcatcc | 59ºC | 550 |
| EXON 10 | PCR and sequencing | Foward: tatgctacggaactgcacca  Reverse: tctgaaatgggaaagcatcc | 60ºC | 600 |
| EXONS 11-13 | PCR and sequencing | Foward: ttgtgcatatctttgagcttctt  Reverse: ttttcaccattttgcctttg | 58ºC | 1250 |
|  | Sequencing | Foward: tggtcattttgcactacctcttt  Reverse: ttcaaagaatccacatgacttga |  |  |
| EXON 14 | PCR and sequencing | Foward: caaagccatcaaaacccaat  Reverse: gggttcccagctagttccat | 59ºC | 550 |
