# Supplementary material for: Association of Single Nucleotide Polymorphisms in the Lens Epithelium-Derived Growth Factor (LEDGF/p75) with HIV-1 Infection Outcomes in Brazilian HIV-1+ Individuals
Source: PLoS One. 2014 Jul 21;9(7):e101780. doi: 10.1371/journal.pone.0101780 (PMC4105638; doi:10.1371/journal.pone.0101780)
Supplement: Table S2 — Primers and probes used for PSIP1 candidate SNPs genotyping. (DOCX) [file pone.0101780.s002.docx]

**Table S2:** TaqMan® assays used for *PSIP1* candidate SNPs genotyping.

| **dbSNP #rs code** | **Base change** | **Chromosome 9 position #** | **Gene position** | **TaqMan Assay ID or custom primers and probes** |
| --- | --- | --- | --- | --- |
| rs17337140 | A/G | 15481190 | intron 5 | C_34288946_10 |
| rs1033056 | A/G | 15483211 | intron 5 | C_2757693_20 |
| rs2737829 | C/G | 15486721 | intron 4 | C_16289866_10 |
| rs10119931 | A/C | 15495078 | intron 2 | C_2757709_20 |
| rs12339417 | C/T | 15498495 | intron 2 | C_31936110_10 |
| rs10283923 | C/G | 15500150 | intron 2 | C_29529242_10 |
| rs10962048 | G/A | 15507808 | intron 1 | Forward: 5’- CCTCTGTAAGCATTATGGTGTTGCA-3’ |
|  |  |  |  | Reverse: 5’-GCCTTTAGACTTCCCTCACAAATCA-3’  5’-VIC-CAGTCATCCT**G**ATGGTC-3’  5’-FAM-CAGTCATCCT**C**ATGGTC-3’ |
| rs7470146 | C/G | 15509366 | intron 1 | C_29339368_10 |
| rs2277191 | A/G | 15510589 | 5’UTR | C_15883595_10 |

# Chromosome positions according to contig NT_008413.18.
