# Supplementary material for: Association of Single Nucleotide Polymorphisms in the Lens Epithelium-Derived Growth Factor (LEDGF/p75) with HIV-1 Infection Outcomes in Brazilian HIV-1+ Individuals
Source: PLoS One. 2014 Jul 21;9(7):e101780. doi: 10.1371/journal.pone.0101780 (PMC4105638; doi:10.1371/journal.pone.0101780)
Supplement: Table S3 — Frequencies of each genotype, allele and minor allele carriers of PSIP1 SNPs and haplotypes in in population controls and HIV+ patients. (DOCX) [file pone.0101780.s003.docx]

**Table S3:** Frequencies of each genotype, allele and minor allele carriers of *PSIP1* SNPs and haplotypes in population controls and HIV^+^ patients.

| **SNP** | **Genotype/allele** | **Controls*** | **HIV^+^ patients*** |
| --- | --- | --- | --- |
| **rs61744944** | **AA** | 169 (0.97) | 152 (0.93) |
|  | **AT** | 5 (0.03) | 10 (0.06) |
|  | **TT** | 0 (0.00) | 1 (0.01) |
|  |  | 174 | 163 |
|  | **A** | 343 (0.99) | 314 (0.96) |
|  | **T** | 5 (0.01) | 12 (0.04) |
|  | **AT+TT** | 5 (0.03) | 11 (0.07) |
|  |  |  |  |
| **rs17337140** | **GG** | 121 (0.74) | 137 (0.85) |
|  | **GA** | 42 (0.26) | 24 (0.15) |
|  | **AA** | 0 (0.00) | 0 (0.00) |
|  |  | 163 | 161 |
|  | **G** | 284 (0.87) | 298 (0.93) |
|  | **A** | 42 (0.13) | 24 (0.07) |
|  | **GA+AA** | 42 (0.26) | 24 (0.15) |
|  |  |  |  |
| **rs1033056**** | **GG** | 56 (0.74) | n.d |
|  | **GA** | 15 (0.20) |  |
|  | **AA** | 5 (0.07) |  |
|  |  | 76 |  |
|  | **G** | 127 (0.84) |  |
|  | **A** | 25 (0.16) |  |
|  | **GA+AA** | 18 (0.24) |  |
|  |  |  |  |
| **rs2737829** | **CC** | 45 (0.85) | 137 (0.93) |
|  | **CG** | 8 (0.15) | 10 (0.07) |
|  | **GG** | 0 (0.00) | 1 (0.01) |
|  |  | 53 | 148 |
|  | **C** | 98 (0.92) | 284 (0.96) |
|  | **G** | 8 (0.08) | 12 (0.04) |
|  | **CG+GG** | 8 (0.15) | 11 (0.07) |
|  |  |  |  |
| **rs10119931** | **AA** | 70 (0.81) | 135 (0.80) |
|  | **AC** | 16 (0.19) | 29 (0.17) |
|  | **CC** | 0 (0.00) | 5 (0.03) |
|  |  | 86 | 169 |
|  | **A** | 156 (0.91) | 299 (0.88) |
|  | **C** | 16 (0.09) | 39 (0.12) |
|  | **AC+CC** | 16 (0.19) | 34 (0.20) |
|  |  |  |  |
| **rs12339417**** | **CC** | 135 (0.82) | n.d |
|  | **CT** | 24 (0.15) |  |
|  | **TT** | 6 (0.04) |  |
|  |  | 165 |  |
|  | **C** | 294 (0.89) |  |
|  | **T** | 36 (0.11) |  |
|  | **CT+TT** | 30 (0.18) |  |
|  |  |  |  |
| **rs10283923** | **CC** | 66 (0.73) | 103 (0.60) |
|  | **CG** | 21 (0.23) | 54 (0.32) |
|  | **GG** | 3 (0.03) | 14 (0.08) |
|  |  | 102 | 171 |
|  | **C** | 153 (0.85) | 260 (0.76) |
|  | **G** | 27 (0.15) | 82 (0.24) |
|  | **CG+GG** | 24 (0.26) | 68 (0.40) |
|  |  |  |  |
| **rs10962048** | **GG** | 83 (0.86) | 147 (0.86) |
|  | **GA** | 13 (0.14) | 21 (0.12) |
|  | **AA** | 0 (0.00) | 2 (0.01) |
|  |  | 96 | 170 |
|  | **G** | 179 (0.93) | 315 (0.93) |
|  | **A** | 13 (0.07) | 25 (0.07) |
|  | **GA+AA** | 13 (0.14) | 23 (0.14) |
|  |  |  |  |
| **rs7470146** | **GG** | 31 (0.46) | 82 (0.48) |
|  | **GC** | 26 (0.39) | 68 (0.40) |
|  | **CC** | 10 (0.15) | 21 (0.12) |
|  |  | 67 | 171 |
|  | **G** | 88 (0.66) | 232 (0.68) |
|  | **C** | 46 (0.34) | 110 (0.32) |
|  | **GC+CC** | 36 (0.54) | 89 (0.52) |
|  |  |  |  |
| **rs2277191** | **GG** | 140 (0.97) | 165 (0.96) |
|  | **GA** | 5 (0.03) | 5 (0.03) |
|  | **AA** | 0 (0.00) | 1 (0.01) |
|  |  | 145 | 171 |
|  | **G** | 285 (0.98) | 335 (0.98) |
|  | **A** | 5 (0.02) | 7 (0.02) |
|  | **GA+AA** | 5 (0.03) | 6 (0.04) |
|  |  |  |  |
| **Haplotype** |  |  |  |
| **rs61744944/ rs17337140/ rs2737829/rs10119931/rs10283923/ rs10962048/rs7470146/rs2277191***** | **A/G/C/A/C/G/G/G** | 0.37 | 0.35 |
|  | **A/G/C/A/C/G/C/G** | 0.29 | 0.32 |
|  | **A/A/C/A/C/G/G/G** | 0.10 | 0.07 |
|  | **A/G/C/A/G/A/G/G** | 0.03 | 0.05 |

*results are shown as N (frequency). **p < 0.05 in HWE test. ***Haplotype frequencies were estimated by maximum likelihood. Haplotypes with frequencies of less than 0.03 were suppressed. n.d: not done.
