# Supplementary material for: Association of Single Nucleotide Polymorphisms in the Lens Epithelium-Derived Growth Factor (LEDGF/p75) with HIV-1 Infection Outcomes in Brazilian HIV-1+ Individuals
Source: PLoS One. 2014 Jul 21;9(7):e101780. doi: 10.1371/journal.pone.0101780 (PMC4105638; doi:10.1371/journal.pone.0101780)
Supplement: Table S4 — Frequency of PSIP1 haplotypes in HIV+ patients and population controls. (DOCX) [file pone.0101780.s004.docx]

**Table S4:** Frequency of *PSIP1* haplotypes in HIV+ patients and population controls.

| **Haplotype** | **rs61744944** | **rs17337140** | **rs2737829** | **rs10119931** | **rs10283923** | **rs10962048** | **rs7470146** | **rs2277191** | **Controls** | **HIV^+^ patients** |
| --- | --- | --- | --- | --- | --- | --- | --- | --- | --- | --- |
| **1** | A | A | C | C | C | A | C | G | 0.00232 | NA |
| **2** | A | A | G | C | C | A | C | G | 0.00305 | NA |
| **3** | A | G | C | A | C | G | C | G | 0.28894 | 0.31741 |
| **4** | A | G | G | A | C | G | C | G | 0.01475 | 0.00000 |
| **5** | A | G | C | C | C | G | C | G | 0.01187 | NA |
| **6** | A | A | C | C | G | A | C | G | 0.00298 | NA |
| **7** | A | A | G | C | G | A | C | G | 0.00312 | NA |
| **8** | A | G | C | A | G | G | C | G | NA | 0.00423 |
| **9** | A | G | C | C | G | G | C | G | 0.00902 | NA |
| **10** | A | A | C | A | C | G | G | G | 0.10403 | 0.07418 |
| **11** | A | G | G | A | C | G | G | A | NA | 0.00323 |
| **12** | A | G | C | A | C | G | G | G | 0.37079 | 0.34854 |
| **13** | A | G | G | A | C | G | G | G | 0.04822 | 0.01376 |
| **14** | A | G | C | C | C | G | G | G | NA | 0.00311 |
| **15** | A | A | G | A | G | A | G | G | 0.00656 | NA |
| **16** | A | G | C | A | G | A | G | A | 0.01779 | NA |
| **17** | A | G | G | A | G | A | G | A | NA | 0.01724 |
| **18** | A | G | C | A | G | A | G | G | 0.02890 | 0.04650 |
| **19** | A | G | G | A | G | A | G | G | NA | 0.00773 |
| **20** | A | G | C | C | G | A | G | G | NA | 0.00316 |
| **21** | A | G | C | A | G | G | G | G | NA | 0.04786 |
| **22** | A | G | G | A | G | G | G | G | 0.00710 | 0.00172 |
| **23** | A | G | C | C | G | G | G | G | 0.06080 | 0.07430 |
| **24** | T | G | C | C | G | G | G | G | 0.00667 | 0.03703 |

SNPs are organized according to chromosomal positions from *PSIP1* 5’ – 3’. Frequencies were estimated by maximum likelihood. NA = not available.
